# Brazilian vegetarians diet quality markers and comparison with the general population: A nationwide cross-sectional study

**DOI:** 10.1371/journal.pone.0232954

**Published:** 2020-05-12

**Authors:** Shila Minari Hargreaves, Wilma Maria Coelho Araújo, Eduardo Yoshio Nakano, Renata Puppin Zandonadi

**Affiliations:** 1 Department of Nutrition, Faculty of Health Sciences, University of Brasilia (UnB), Brasilia, Brazil; 2 Department of Statistics, University of Brasilia, Brasilia, DF, Brazil; State University of Rio de janeiro, BRAZIL

## Abstract

**Background:**

Vegetarianism is an increasingly common practice worldwide. Despite good evidence from other countries regarding vegetarians’ diet quality, data from the Brazilian population is still scarce.

**Objective:**

To characterize the vegetarian Brazilian population and evaluate their diet quality compared to the general Brazilian population.

**Methods:**

We performed a nationwide cross-sectional study using an online self-administered questionnaire, previously validated for the Brazilian population, to evaluate diet quality markers of vegetarians. The invitation to participate in the survey was spread nationwide, aimed at vegetarian communities. Individuals who considered themselves vegetarians and were at least 18 years old were eligible to participate. The results on regular intake and intake adequacy were compared among vegetarians and between genders using the Pearson’s chi-square test. The body mass index (BMI) were analyzed by the Analysis of Variance (ANOVA) followed by Tukey *post-hoc* test. The Kolmogorov-Smirnov test verified normality. All analyses considered bilateral hypotheses and a significance level of 5% (p <0.05).

**Results:**

Brazilian vegetarians presented better diet quality markers, such as higher regular weekly intake and adequate daily intake of fruits and vegetables, and lower regular intake of soft drinks when compared to the general Brazilian population. Vegetarians also presented a proportionally higher consumption of natural foods and lower consumption of processed foods. Among vegetarians, a higher proportion of vegans showed positive results regarding diet markers analysis, when compared to vegetarians, pesco-vegetarians, and semi-vegetarians.

**Conclusions:**

Vegetarians showed better results of diet adequacy when compared to the general population in Brazil, and vegans fared better when compared with other vegetarians. Despite the good results found, a large proportion of the participants still did not achieve the fruits and vegetables daily intake, according to the World Health Organization recommendations.

## Introduction

Vegetarianism is a broad concept that encompasses various dietary patterns, according to how restricted the consumption of animal products is in one’s diet [1]. Around the world, the prevalence of vegetarians varies considerably, ranging from 40% in India, the most representative country, to only 1% in Portugal [2], or 3.3% in the United States [3]. According to data from the Brazilian Institute of Public Opinion and Statistics (IBOPE), 14% of the overall Brazilian population are classified as vegetarians [4].

Different factors can influence the adoption of a vegetarian diet. Ethical reasons are generally considered to be the primary motivators, being related to the concept that exploiting animals for consumption is not morally correct [2,5]. Health improvement is also cited as one of the reasons why people choose to become vegetarians, as well as environmental concerns regarding meat production impact on natural resources. Belonging to a religion which encourages meat avoidance, such as Hinduism and Adventism, is also an important factor that leads to the adoption of a vegetarian diet [5,6].

There are different types of vegetarian diets, classified according to whether or not other animal products, such as eggs or dairy products, are present in the diet, as well as other parameters related to the origin of food and its preparation. The two main diet categories are vegetarian (including ovo-lacto-vegetarian, pesco-vegetarian, and semi-vegetarian) and vegan (excludes any food of animal origin, as well as other non-food products such as clothing and cosmetics, which may contain animal-derived products) [7,8]. In this regard, it is essential to mention that very restrictive diets may pose a higher risk of nutritional deficiencies, with potentially deleterious health effects [9–11].

On the other hand, vegetarian diets have already been associated with better health outcomes, such as prevention of cardiovascular diseases, due to its effectiveness in reducing total and LDL serum cholesterol [12,13]; reduced risk and better control of type 2 diabetes, by improving insulin sensitivity and reducing oxidative stress serum markers [14]; reduced risk of some types of cancer [13,15]; and better mood and lower levels of stress and anxiety [16,17].

A relevant factor that contributes to better overall health is the lower average BMI observed in vegetarians, which in turn are associated with a lower risk of metabolic syndrome [13,14,18,19]. Another potential mechanism that explains better health outcomes in vegetarians is the potential benefit that their diet has on the gut microbiome. Improving gut health can, in turn, result in reduced inflammation, cardiovascular disease prevention, better insulin sensitivity, improvement of blood lipid levels, more satiety, obesity control, and colorectal cancer [20–23]. All these health benefits observed in vegetarians are associated with higher intake of fruits, vegetables, nuts and whole grains (foods rich in fiber and phytochemicals), lower caloric intake, lower saturated fat, and proportionally higher mono and poly-unsaturated fats intake, and a higher proportion of vegetable protein compared to animal protein [9,14,24–26].

In order to understand how a dietary pattern potentially influences health, it is necessary to use tools to assess the quality of a studied population’s diet. Diet quality is a concept often adopted in the scientific literature to refer to the eating habits of a population, and it is usually used to describe the individuals’ compliance with dietary recommendations [27]. For this purpose, food surveys are conducted using different methodologies to either evaluate an individual's diet adequacy or to define if the individual’s dietary patterns can ensure health. [27]. Defining the best method to evaluate diet quality can be a challenging task. Using single components of the diet, such as percent of calories from fat or specific nutrients intake, can lead to misclassifications of overall diet quality [28,29]. Research has shown that using shorter questionnaires that target key diet components to generally analyze diet quality (instead of considering the intake of each nutrient alone) may be a more feasible and representative way to evaluate diet quality of a population under study [30–32].

The diet quality of vegetarians has been assessed in previous studies with different instruments and methodologies [7,26,33]. However, despite good evidence from other countries, scientific literature about vegetarian and vegan diet quality in Brazil is still scarce [34,35]. Moreover, to the best of our knowledge, no study focused on globally analyzing the diet quality of different categories of vegetarian diets in comparison with the general population has yet been published in Brazil. Assessing the diet quality of Brazilian vegetarians and vegans in a global and standardized way is fundamental, as it will provide more accurate data on which to base the conduct of health professionals, the development of public policies, and even market reactions to vegetarianism. Therefore, this study aimed to characterize the vegetarian population and evaluate the diet quality of vegetarians in Brazil, comparing among different types of vegetarian dietary patterns, as well as with the general population in the country.

## Materials and methods

### Study design

We carried out a cross-sectional study in three stages: (i) selection of an instrument to evaluate the dietary markers of vegetarians in Brazil; (ii) evaluation of the instrument for online application; (iii) nationwide online application of this instrument among Brazilian vegetarians and vegans.

### Questionnaire

The first part of the instrument included variables that are commonly used to describe populations. We used data from the Brazilian National Institute of Geography and Statistics (Instituto Nacional de Geografia e Estatística—IBGE) as a reference. The selected variables were gender, age, income, schooling level, and housing area [36].

Self-reported weight and height were included to allow an estimation of participants’ body mass index (BMI), which was then used to classify their nutritional status (BMI: Low-weight: <18.5 kg/m^2^; Normal weight: 18.5–24.9 kg/m^2^; Overweight: 25.0–29.9 kg/m^2^; Obesity: ≥ 30 kg/m^2^) [37]. Despite being an indirect method, self-reported weight and height has good compliance and validation among adults and is considered a feasible tool in studies where it is not possible to apply direct measurement [38]. Moreover, we included a question asking the type of vegetarian diet adopted to allow comparisons among different types of vegetarians. For this purpose, we categorized diets using the same classification used in other studies [1,7,8] as (a) vegan (excludes all kinds of animal products); (b) vegetarian (excludes all meats, but allows eggs and/or dairy products); (c) pesco-vegetarians (excludes all meats except for fish); (d) semi-vegetarian (allows meat no more than once a week). Finally, participants were asked about the main motivation for the adoption of a vegetarian diet. Since the range of possibilities is wide, we used the most common motivations cited by other studies [2,5,6,39,40]: (a) ethic/moral; (b) personal health; (c) religion/beliefs/spirituality; (d) environmental impact; (e) aversion/intolerance/allergy; and (f) others.

We used the Food questionnaire of a survey conducted by the Brazilian Ministry of Health to evaluate nutritional quality [41]. The “Surveillance System of Risk and Protection Factors for Chronic Diseases by Telephone Inquiry” (*Vigitel*) is a large population study conducted annually by the Ministry of Health since 2006, and it is part of a set of actions aimed at the control of chronic non-communicable diseases. This instrument, also used in the present study, enables the identification of the consumption of healthy and unhealthy foods, factors considered relevant to evaluate the nutritional quality of the Brazilian diet. It is based on various simplified models used to monitor risk factors for chronic diseases and has been improved over the years up to the latest version. The food questionnaire includes questions on how often respondents consume beans, fruits, raw and cooked vegetables, milk, meats, and soft drinks (sugary and non-sugary soft drinks) [42]. There is no question regarding vegetarianism on *Vigitel*, as it is a broad tool to evaluate diet quality. This tool has already been validated for the Brazilian population [43,44] (which is composed in its majority by omnivores) and used across the whole country every year since 2006, with a probabilistic nationwide sample selection of individuals. Therefore, it enables the assessment of the nutritional quality of the studied vegetarian/vegan population, as well as their comparison with the general Brazilian population.

The dietary markers evaluated by the *Vigitel* questionnaire were: (a) fruit regular intake; (b) vegetables regular intake; (c) raw vegetables regular intake; (d) cooked vegetables regular intake; (e) fruits and vegetables regular intake; (f) fruits and vegetables adequate daily intake; and (f) regular soda intake (a negative dietary marker). Regular intake refers to the consumption of the item in at least five days per week. Results were presented as the percentage of individuals classified positively according to each item, as described by the Ministry of Health guidelines for *Vigitel* analysis [45].

The *Vigitel* questionnaire also includes questions related to specific food items consumption, for which the participants should answer if they consumed or not one of those foods on the previous day. One of the questions was related to natural/fresh foods and the other one, to processed/industrialized foods. Each item contained from one to six foods considered as being on the same category. Examples of categories for natural foods are cereals, legumes, green vegetables, yellow/orange fruits. The item related to green vegetables, for example, was “Lettuce, kale, broccoli, cress or spinach”. Similarly, processed foods were separated into similar items. An example of an item was “instant noodles/soup, frozen lasagna or other frozen instant meals”, related to the category of ready-to-eat meals. In total, there were 12 items related to natural foods and 13 items related to processed foods. The result was described as the average of items marked as consumed, among the list of items, by the participants of the study. Consumption frequency for each item was also described for both categories (natural and processed foods), in order to evaluate how much each item contributed to the food intake from the previous day. The results were presented as the mean frequencies of consumption for each item.

### Evaluation of the instrument for online application

Since the questionnaire used for *Vigitel* research is conducted by phone, it was necessary to evaluate its use in a self-administered online format. For this step, 20 individuals were conveniently selected to answer the same questionnaire twice, on the same day with at least a two-hour interval, and without previous knowledge that it would be necessary to answer the questions for the second time. Answers had to be collected on the same day since some of the questions were related to food intake on the previous day. We divided the sample into two groups of ten individuals, according to the order of replies: (a) first time online self-administered and second time by phone interview; and (b) first time by phone interview and second time online self-administered. The groups answered both versions in alternate ways to avoid a possible confounding learning effect. We sent the online self-administered version through a link with the questionnaire, created on the SurveyMonkey® platform. The questions used in the online form were the same as the ones asked by phone interview (S1 and S2 Tables). The authors of this study conducted the phone call interview. An example of question (taken from the *Vigitel* original questionnaire) is: “How many times per week do you usually eat fruits?” and the possible answers are: “once to twice per week”, “three to four times per week”, “five to six times per week”, “every day, including weekends”, “almost never” and “never”. After this step, the answers were analyzed to evaluate and compare *intra-* and *inter-*group discordance. The online application would be considered valid if its values were equivalent to the standard application over the phone. The equivalence of these results was measured through full agreement and agreement measures. In this sense, the concordance between the telephone and online responses was verified using Cohen’s kappa coefficient (for categorical variables) and by the intraclass correlation coefficient—ICC (for quantitative variables).

### Procedures

After the questionnaire had been evaluated for online self-administration, we conducted a cross-sectional online survey in 2018. Vegetarian adults (over 18 years of age) from the entire country were recruited to participate in the study. The questionnaire was applied using the SurveyMonkey® tool. We sent participants a web link to access the survey via email, messaging applications, and social networks. Volunteers were recruited nationwide with the help of vegetarianism support groups and social media to reach as many vegetarians as possible. The “Brazilian Vegetarian Society” helped to publicize the study through their mailing list, as well as by announcing it at a congress aimed at the vegetarian population. Health professionals recognized as big influencers of vegetarianism in Brazil also helped to publicize the study through social media channels. Volunteers received, together with the research link, an invitation to participate, as well as the Consent Form. The study was approved by the Research Ethics Committee from the Health Institute of the University of Brasília (protocol number: 94114118.7.0000.0030) and conducted according to the guidelines laid down in the Declaration of Helsinki.

Results of the sample size and distribution by states were compared with data from MapaVeg (an independent project which produces content targeted at the vegetarian population) to evaluate if the sample was representative of the Brazilian vegetarian population. No official data with vegetarian population distribution had been published in Brazil, being the MapaVeg poll the most consistent and reliable data available. Therefore, we based the sampling plan on MapaVeg data (n = 29,282) [46]. The sampling size was calculated considering an error (e) of 3% and a level of significance (α) of 5%, as described by Hair & cols. [47], in which the minimum sample was estimated in 1,030 patients.

The categorical variable descriptions were presented as frequencies and percentages; the quantitative variables were described regarding mean and standard deviation. The results on regular intake and intake adequacy were compared among vegetarians and between genders through the Pearson’s chi-square test. In the case of BMI, this comparison was performed using the Analysis of Variance (ANOVA) followed by Tukey *post-hoc* test. The Kolmogorov-Smirnov test verified normality.

The diet quality results obtained in this study for vegetarians were compared with data from the last published version of the general Brazilian population study (*Vigitel*) [48]. This comparison was carried out to evaluate if there were differences in diet quality between vegetarians and the general Brazilian population. Results were considered statistically different if the confidence intervals did not overlap. The confidence intervals of the percentages were calculated by normal approximation. No test was performed to compare the two groups because the sampling method was different (stratified sampling for the Brasilian population versus convenience sampling for the vegetarian population). All analyses considered bilateral hypotheses and a significance level of 5% (p <0.05). The analyses were performed by the SPSS (Statistical Package for Social Science) and Microsoft Excel.

## Results

The questionnaire had nationwide representation with a sample from all 27 Brazilian states (S3 Table and S1 Fig). It was possible to obtain a representative sample of Brazil’s vegetarian population, with a similar distribution by states as observed in a national online poll conducted by the MapaVeg project, which had over 29 thousand participants [46]. By comparing the study sample with the data presented by MapaVeg, we noticed that the only state that presented a divergence in the proportion was Distrito Federal (DF). This result was attributed to the fact that the study started in this state and therefore reached more individuals in this area. On the other hand, in all other Brazilian States, the proportions were similar. Moreover, the minimum sample size estimated was 1,030 individuals (with a 3% error chance and a confidence interval of 95%), and we obtained in the study a sample of 3,319 participants.

### Population characteristics

The majority (65.0%) were female, and 65.3% of individuals were below 40 years old. Regarding the type of diet, ovo-lacto-vegetarians were the most prevalent, followed by vegans. Pesco-vegetarian and semi-vegetarian diets had lower prevalence, accounting together for 13.4% of the sample. The primary motivation for adopting a vegetarian diet was ethical/moral reasons (61.9% of the sample), followed by environmental impact (11.8%) and personal health (11.4%) (Table 1). Nutritional status, classified by BMI, showed that 65.8% of individuals were eutrophic (BMI between 18.5 and 24.9kg/m2). The sociodemographic characteristics of the population are presented in Table 1.

**Table 1:** Sociodemographic characteristics of the studied population (N = 3,319).

Characteristic	Category	Respondents (N = 3319)	
		Number	Percentage
**Gender**	Male	1163	35.0%
	Female	2156	65.0%
**Age**	18–24	919	27.7%
	25–29	577	17.4%
	30–39	684	20.6%
	40 or more	1139	34.3%
**Housing location**	Capital or metropolitan area	2243	67.6%
	Urban area (other cities)	993	29.9%
	Rural area	83	2.5%
**Average income**^**a**^	Less than two minimum wages^b^	490	14.8%
	Between two and five minimum wages	971	29.2%
	Between five and ten minimum wages	910	27.4%
	Between ten and twenty minimum wages	545	16.4%
	Above twenty minimum wages	212	6.4%
	Not informed	191	5.8%
**Educational level**	No education	0	0%
	Elementary School, incomplete	2	0.1%
	Elementary School, complete	6	0.2%
	High School, incomplete	34	1.0%
	High School, complete	359	10.8%
	University level, incomplete	825	24.9%
	University level, complete	2093	63.1%
**Type of diet**	Vegan	1115	33.6%
	Ovo-lacto-vegetarian	1601	48.2%
	Pesco-vegetarian	232	7.0%
	Semi-vegetarian	371	11.2%
**Main motivation for adopting the diet**	Ethics/moral	2053	61.9%
	Personal health	380	11.4%
	Religion/beliefs/spirituality	152	4.6%
	Environmental impact	392	11.8%
	Aversion/intolerance/allergy	121	3.6%
	Others	221	6.7%
**Time adopting the diet**	Less than one year	764	23.0%
	Between one and five years	1505	45.3%
	More than five years	1050	31.6%
**Nutritional status**	Underweight (<18.5kg/m^2)^	189	5.7%
[37]	Adequate (18.5–24.9kg/m^2^)	2184	65.8%
	Overweight (25–29.9kg/m^2^)	687	20.7%
	Obese (≥30kg/ m^2^)	235	7.1%
	Not informed	24	0.7%

* Different capital letters mean that there was a statistical difference among vegetarian groups (p<0.05).

^a^ One minimal wage was equivalent to $248.00 (on 12.31.2018).

^b^ Minimum wage: according to the Brazilian Constitution, it corresponds to the minimum necessary income to fulfill all the essential vital needs (housing, food, education, health, leisure, clothing, hygiene, transportation, and social security).

### Evaluation of the instrument to online self-report application

Results from the instrument evaluation step for online application showed absolute good agreement for the answers comparison. Kappa was statistically significant (p<0.05) for all analyses, when comparing the answers given online and by phone interview, except for the item “regular fruit intake”, in which Kappa did not reach statistical significance. However, the absolute agreement for the same item was 85%, considered high [49]. For the item “regular soft drink intake”, it was not possible to calculate kappa because the answers were equal for all the participants in one of the methods. The absolute agreement, however, was 95%. Therefore, the feasibility of the questionnaire online application was confirmed since the results were very similar to the ones obtained with the phone interview method.

### Diet quality regarding the adequacy to the Brazilian guideline

As presented in Table 2, 60.1% of the respondents reported regular consumption of fruits and vegetables, considering all the vegetarian categories. The highest frequency was observed among vegans compared to the other three categories. When the recommended daily intake of fruits and vegetables was analyzed, 38.1% of the study population achieved the recommendations (Table 2). In this aspect, vegans also had better levels compared to the other. No statistical difference was found among the three other categories.

**Table 2:** Diet adequacy, by gender and type of diet (N = 3,319).

		Gender	
	Total*	Male	Female
	Number (%; 95% CI)	Number (%; 95% CI)	Number (%; 95% CI)
Regular intake of vegetables^1^			
Vegans	1024 (91.8%; 90.2–93.4%)^A^	381 (91.6%; 88.9–94.3%)	643 (92.0%; 90.0–94.0%)
Ovo-lacto-vegetarians	1362 (85.1%; 83.3–86.8%)^B^	469 (84.7%; 81.7–87.7%)	893 (85.3%; 83.1–87.4%)
Pesco-vegetarians	199 (85.8%; 81.2–90.3%)^B^	39 (79.6%; 67.9–91.3%)	160 (87.4%; 82.6–92.3%)
Semi-vegetarians	279 (75.2%; 70.8–79.6%)^C^	111 (77.1%; 70.1–84.0%)	168 (74.0%; 68.3–80.0%)
Total**	**2864 (86.3%; 85.1–87.5%)**	**1000 (86.0%; 84.0–88.0%)**^**a**^	**1864 (86.5%; 85.1–87.9%)**^**a**^
Regular intake of raw vegetables^1^			
Vegans	723 (64.8%; 62.0–67.7%^A^	261 (62.7%; 58.1–67.4%)	462 (66.1%; 62.6–69.6%)
Ovo-lacto-vegetarians	910 (56.8%; 54.4–59.3%)^AB^	298 (53.8%; 49.6–58.0%)	612 (58.5%; 55.5–61.4%)
Pesco-vegetarians	137 (59.1%; 52.7–65.4%)^AB^	23 (46.9%; 32.5–61.4%)	114 (62.3%; 55.2–69.4%)
Semi-vegetarians	204 (55.0%; 49.9–60.1%)^B^	72 (50.0%; 41.7–58.3%)	132 (58.1%; 51.7–64.6%)
Total**	**1974 (69.5%; 57.8–61.1%)**	**654 (56.2%; 54.6–70.9%)**^**a**^	**1320 (61.2%; 59.2–63.3%)**^**b**^
Regular intake of cooked vegetables^1^			
Vegans	787 (70.6%; 67.9–73.2%)^A^	259 (62.3%; 57.6–66.9%)	528 (75.5%; 72.3–78.7%)
Ovo-lacto-vegetarians	998 (62.3%; 60.0–64.7%)^B^	299 (54.0%; 49.8–58.1%)	699 (66.8%; 63.9–69.6%)
Pesco-vegetarians	128 (55.2%; 48.7–61.6%)^BC^	20 (40.8%; 26.6–55.1%)	108 (59.0%; 51.8–66.2%)
Semi-vegetarians	183 (49.3%; 44.2–54.4%)^C^	71 (49.3%; 41.0–57.6%)	112 (49.3%; 42.8–55.9%)
Total**	**2096 (63.2%; 61.5–64.8%)**	**649 (55.8%; 52.9–58.7%)**^**a**^	**1447 (67.1%; 65.1–69.1%)**^**b**^
Regular intake of fruits^1^^,^ ^2^			
Vegans	844 (75.7%; 73.2–78.2%)^A^	289 (69.5%; 65.0–73.9%)	555 (79.4%; 76.4–82.4%)
Ovo-lacto-vegetarians	969 (60.5%; 58.1–62.9%)^B^	277 (50.0%; 45.8–54.2%)	692 (66.1%; 63.2–69.0%)
Pesco-vegetarians	147 (63.4%; 57.1–69.6%)^B^	26 (53.1%; 38.6–67.5%)	121 (66.1%; 59.2–73.0%)
Semi-vegetarians	224 (60.4%; 55.4–65.4%)^B^	85 (59.0%; 50.9–67.2%)	139 (61.2%; 54.9–67.6%)
Total**	**2184 (65.8%; 64.2–67.4%)**	**677 (58.2%; 55.4–61.1%)**^**a**^	**1507 (69.9%; 68.0–71.8%)**^**b**^
Regular intake of fruits and vegetables^3^			
Vegans	797 (71.5%; 68.8–74.1%)^A^	275 (66.1%; 61.5–70.7%)	522 (74.7%; 71.5–77.9%)
Ovo-lacto-vegetarians	878 (54.8%; 52.4–57.3%)^B^	249 (44.9%; 40.8–49.1%)	629 (60.1%; 57.1–63.1%)
Pesco-vegetarians	132 (56.9%; 50.5–63.3%)^B^	23 (46.9%; 32.5–61.4%)	109 (59.6%; 52.4–66.7%)
Semi-vegetarians	189 (50.9%; 45.8–56.1%)^B^	71 (49.3%; 41.0–57.6%)	118 (52.0%; 45.4–58.5%)
Total**	**1996 (60.1%; 58.5–61.8%)**	**618 (53.1%; 50.3–56.0%)**^**a**^	**1378 (63.9%; 61.9–65.9)**^**b**^
Regular intake of soft drinks^1^			
Vegans	16 (1.4%; 0.7–2.1%)^A^	11 (2.6%; 1.1–4.2%)	5 (0.7%; 0.1–1.3%)
Ovo-lacto-vegetarians	93 (5.8%; 4.7–7.0%)^B^	40 (7.2%; 5.1–9.4%)	53 (5.1%; 3.7–6.4%)
Pesco-vegetarians	5 (2.2%; 0.3–4.0%)^A^	0 (0%; —)	5 (2.7%; 0.4–5.1%)
Semi-vegetarians	14 (3.8%; 1.8–5.7%)^AB^	4 (2.8%; 0.1–5.5%)	10 (4.4%; 1.7–7.1%)
Total**	**128 (3.9%; 3.2–4.5%)**	**55 (4.7%; 3.5–6.0%)**^**a**^	**73 (3.4%; 2.6–4.2%)**^**a**^
Adequate daily intake of fruits and vegetables^4^			
Vegans	551 (49.4%; 46.5–52.4%)^A^	172 (41.3%; 36.6–46.1%)	379 (54.2%; 50.5–57.9%)
Ovo-lacto-vegetarians	523 (32.7%; 30.4–35.0%)^B^	149 (26.9%; 23.2–30.6%)	374 (35.7%; 32.8–38.6%)
Pesco-vegetarians	84 (36.2%; 30.0–42.4%)^B^	14 (28.6%; 15.5–41.7%)	70 (38.3%; 31.1–45.4%)
Semi-vegetarians	108 (29.1%; 24.5–33.8%)^B^	36 (25.0%; 17.8–32.2)	72 (31.7%; 25.6–37.8%)
Total**	**1266 (38.1%; 36.5–39.8%)**	**371 (31.9%; 29.2–34.6%)**^**a**^	**895 (41.5%; 39.4–43.6%)**^**b**^

^1^ Percentage of individuals who consume it five or more days per week.

^2^ Considers intake of fruits or fresh fruit juices.

^3^ Percentage of individuals who consume fruits and vegetables five or more days per week.

^4^ Percentage of individuals who eat five or more portions per day.

* Different capital letters mean that there was a statistical difference among vegetarian groups (p<0.05).

** Different small letters mean that there was a statistical difference between genders (p<0.05).

Soft drinks intake was considered a marker of an unhealthy diet. Carbonated drinks and artificial juices were included in the category of soft drinks. Results showed an average of only 3.9% frequent consumption (five or more days per week) among the whole study population (Table 2), with the lowest intake among vegans and pesco-vegetarians, and highest among ovo-lacto-vegetarians. Semi-vegetarians had intermediate values (Table 2).

We also evaluated the consumption of vegetables (any kind), raw vegetables, cooked vegetables, and fruits (including fruits and fresh fruit juices). Results showed the same tendency as previous analyses, with vegans showing the highest proportion of regular intake. Regarding gender, females had a statistically higher regular intake of raw and cooked vegetables and fruits. Women also had higher regular weekly intake and adequate daily intake of fruits and vegetables compared to men (Table 2).

The questionnaire used in the research included two questions related to the intake of specific foods, and individuals had to refer if they consumed or not the foods the day before. One question was related to natural foods, and the second question, to industrialized products. For each item, if the individual ate at least one of the foods on the list, they would answer YES. In total, 12 items composed the list of natural foods, and 13 items, the list of industrialized products. Table 3 shows the average number of items marked in each category on the previous day.

**Table 3:** Relative consumption of natural foods and processed items on the previous day, by type of diet. N = 3,319.

	Natural Foods (n = 12)		Industrialized products (n = 13)	
	%*	95% CI**	%	95% CI**
Vegans	57.5% ^A^	56.8–58.2%	13.0% ^A^	12.2–13.8%
Ovo-lacto-vegetarians	57.8% ^A^	57.0–58.6%	19.4% ^B^	18.6–20.1%
Pesco-vegetarians	58.3% ^A^	56.2–60.3%	16.4% ^C^	14.4–18.3%
Semi-vegetarians	62.2% ^B^	60.4–64.0%	20.9% ^B^	19.3–22.5%
Total	58.2%	57.7–58.7%	17.2%	16.7–17.7%

* Different capital letters mean that there was a statistical difference among vegetarian groups (p<0.05).

** 95% Confidence Intervals.

**Fig 1:**
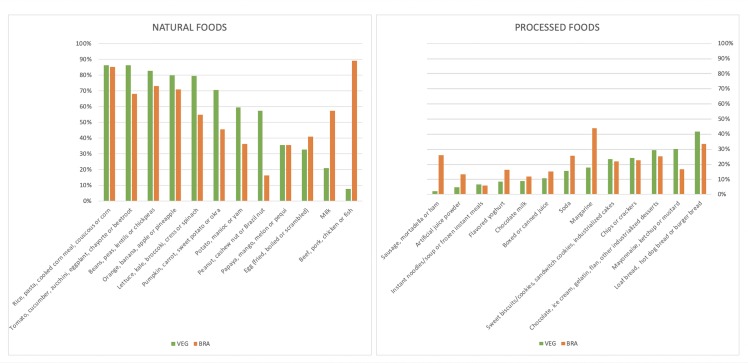
Average percentage of individuals who consumed different groups of foods on the previous day. Comparison of data from this study and the Brazilian general population [50]. VEG: study participants; BRA: Brazilian general population.

Single item analysis showed that, from the 12 natural foods categories, the ones with higher consumption (consumed by more than 75% of the individuals) were: cereals, legumes, two vegetable items, and one fruit item. The less consumed items were meats, milk, and eggs. Regarding processed foods, the most consumed item was bread (loaf bread, whole-grain loaf bread, hot dog bread, and hamburger bread), followed by sauces (mayonnaise, ketchup, and mustard), and the desserts item (chocolate, ice cream, gelatin, flan, and other industrialized desserts). The less consumed items were processed meats, artificial juice powder e ready-to-eat meals (instant noodles/soups, frozen lasagna, and other frozen ready-to-eat meals). Data is shown in Fig 1. The complete list of items is described in S4 Table.

The results obtained in this study were also compared to data of the general Brazilian population from *Vigitel* (2018) [50]. In 2018, the *Vigitel* sample size was of 52,395 individuals. Table 4 summarizes the results of the comparison, showing that, on average, vegetarians (considering all categories together) had lower overweight and obesity rates, higher regular intake and adequate daily intake of fruits and vegetables, and lower regular intake of soft drinks when compared to the rest of the Brazilian population. S2 Fig shows the graphic comparison of both groups stratified by age and gender.

**Table 4:** Comparison between results of diet adequacy and prevalence of excess weight and obesity from this study with the Brazilian general population from *Vigitel* 2018 study.

	Vegetarians (all) N = 3,319		Brazilian population* N = 52,395	
	%	95% CI**	%	95% CI**
Excess weight***	27.8%	26.3–29.3%	55.7%	54.8–56.6%
Obesity	7.1%	6.2–8.0%	19.8%	19.1–20.5%
Regular intake of fruits and vegetables	60.1%	58.5–61.8%	33.9%	33.0–34.7%
Regular intake of soft drinks	3.9%	3.2–4.5%	14.4%	13.6–15.1%
Adequate daily intake of fruits and vegetables	38.1%	36.5–39.8%	23.1%	22.4–23.9%

*Vigitel (“Surveillance System of Risk and Protection Factors for Chronic Diseases by Telephone Inquiry”) is a population study conducted annually by the Ministry of Health. Data from *Vigitel* ^50^

** 95% Confidence Intervals.

***Excess weight is the sum overweight and obesity rates.

Comparison from the results of the natural and processed foods intake from the general Brazilian population and our sample showed a small but significant difference. Regarding natural foods intake, the average proportion of items marked by the general population was 56.1% (CI: 55.9–56.2%), compared to 58.2% (CI: 57.7–58.7%) in our study. On the other hand, processed food intake was higher in the Brazilian general population [21.4% (CI: 21.3–21.5%)], when compared to our study data [17.2% (16.7–17.7%)]. Despite the small difference when compared to our sample, the comparison with the vegan group showed a higher contrast. The average number of marked items for vegans in the category of processed foods was 13.0% (12.2–13.8%), which represents around one less item marked, from a list of 13 items (1.69 items versus 2.78 items).

When each item was analyzed separately, is it possible to notice that vegetarians had a higher or similar average intake of all the natural groups except the ones related to animal products (eggs, dairy, and meats), which was higher in the Brazilian general population, as expected. Regarding processed foods, vegetarians had a higher intake of bread and sauces (mayonnaise, ketchup, mustard), while the Brazilian general population had a higher average intake of margarine, soft drinks (soda, artificial juice powder and boxed juice), as well as animal products such as flavored yogurt and processed meats. All the results are presented in Fig 1.

## Discussion

In this study, we evaluated important diet quality markers of Brazilian vegetarians, as well as made comparisons among different types of diets, with a representative sample of the vegetarian population in Brazil. The selected questionnaire also allowed us to make comparisons with data available on the general population in Brazil, measured with the same instrument. The use of the online application of a self-administered tool allowed us to reach a higher number of participants and it is less costly, requires less labor, and less time consuming for the researchers and the participants, compared to the interview by phone.

The Brazilian Ministry of Health’s report with data from the last *Vigitel* classified 55.7% of individuals as being overweight (BMI ≥25kg/ m2), with 19.8% of those considered obese (BMI ≥30kg/ m2) [50]. Our study found a lower prevalence of overweight (27.8%) and obesity (7.1%) than in the general Brazilian population. A Brazilian cross-sectional survey conducted with 50 vegetarians in one Brazilian state found similar results than ours, with a prevalence of 26% overweight and 6% obesity. Different from our study, the authors directly weighted and heightened the participants; however, the sample was smaller than ours [34].

A meta-analysis study that evaluated multiple health outcomes associated with vegetarian and vegan diets, with a total sample of over 130,000 vegetarians and 15,000 vegans, showed that BMI levels were lower among vegetarians and vegans when compared to omnivores. This result is linked to the lower energy intake, usually reported by these populations [13]. The EPIC-oxford study found that average energy intake in vegans was 14% lower when compared to omnivores, with ovo-lacto-vegetarians and pesco-vegetarians having intermediate values [26]. Besides caloric intake, the macronutrient composition of the diet could also play a role in body weight management. A randomized controlled trial assessing the effect of a vegan, low-fat diet to control body weight and insulin resistance showed that the lower fat intake, especially of saturated and trans-fat, associated with proportionally higher polyunsaturated fat intake, was associated with lower body mass in the participants following the vegan diet [51].

Moreover, it has been shown already that vegans present higher consumption of fibers than omnivores, a factor that can also be associated with better weight management and obesity prevention [52]. Clarys et al. (2014) also found lower energy intake in vegans when compared to omnivores, with the less restrictive vegetarian categories showing intermediate levels of daily caloric intake. Lower BMI levels observed in vegetarians when compared to the general population can be related to better health outcomes, since obesity has been linked to higher rates of cardiovascular disease, hypertension, type 2 diabetes mellitus, hyperlipidemia, certain cancers, psychological disorders, among other conditions [53].

Diet adequacy was based on markers of healthy and unhealthy eating habits. The consumption of fruits and vegetables was used as a healthier indicator, and it was considered adequate when the regular frequency was at least five times per week. We observed regular consumption of fruits and vegetables in 60.1% of the individuals. According to data from the last *Vigitel*, the general Brazilian population has much lower levels of fruit and vegetable intake, with an average of 33.9% [50].

Besides evaluating regular frequency (based on weekly consumption), daily intake of fruits and vegetables was also taken into account to evaluate diet adequacy. According to the World Health Organization (WHO), at least 400g of fruits and vegetables (equivalent to five portions) should be ingested daily to achieve adequate intake [54]. The questions about the intake of fruits, natural fruit juices, raw and cooked vegetables were taken into account to calculate the number of daily portions. Fruits could count to a maximum three of the five recommended portions, and fruit juices, only up to one. Vegetables (raw or cooked) could count up to four of the five portions. Only individuals who consumed these foods five or more times per week were included in this estimate.

When the recommended intake of five daily portions was considered, only 23.1% of adequacy was observed in the Brazilian population, according to *Vigitel* 2018 [50]. In contrast, our study found a statistically higher result, with 38.1% of the individuals reporting adequate daily intake, according to WHO recommendations. A review study on vegetarian and low-meat diets showed that, indeed, vegetarian diets are characterized by the higher consumption of fruits, vegetables, and nuts, a factor that is associated with its protective effect against chronic diseases. One possible reason for vegetarians' better adequate intake of fruits and vegetables is the known fact that one of the motivations for adopting a vegetarian diet, besides ethical, religious, or environmental reasons, is health improvement, either as disease prevention or treatment [2,55]. As Lea, Crawford, & Worsley [56] have described, there is a high level of awareness of the health benefits of eating a plant-based diet, such as a vegetarian diet. Therefore, it would be expected that individuals who are willing to change their eating patterns for health purposes would tend to make more conscious food choices, like eating more fruits and vegetables or avoiding soft drinks.

Another factor that might positively influence the diet quality in vegetarians is the concern that a more restricted diet could cause nutrition deficiencies [57]. In this case, despite the motivation to adopt the diet, people who decide to turn vegetarians would put more effort into following a healthier eating pattern to avoid such deficiencies, especially in the case of vegans, which restrict a wider variety of foods. A study conducted in the United States showed that vegans had an overall healthy lifestyle, including good eating habits and regular exercise practice, regardless of the motivation for the adoption of the diet [58].

Regular intake of soft drinks was used as an indicator of unhealthy eating habits. A meta-analysis of 88 studies showed that soft drinks consumption is associated with higher energy intake, elevated BMI, and increased risk of diabetes and other medical outcomes. Furthermore, regular intake of soft drinks can be a marker of poor nutrition, as it has been related to lower intake of fruits and fiber, and higher intake of fast foods and carbohydrates with higher glycemic index [59]. The average percentage of individuals regularly consuming soft drinks was 3.9% in our study, a number significantly lower than the prevalence of 14.6% in the Brazilian population [50]. Data from the previous editions of *Vigitel* showed that the prevalence of regular soft drinks intake has been dropping, from 30.9% in 2007 to 16.5% in 2016, which is considered an improvement in nutritional quality. However, the Brazilian Ministry of Health still considered the prevalence as high and set a goal to reduce this number to 12.5% by 2019 [60]. By observing the trends from the last 11 years and the established projections for the general Brazilian population, it is possible to consider that the prevalence found in our study is very low among vegetarians, even when comparing the different categories separately, with the semi-vegetarians showing the highest prevalence among them (6.0%). This result could again be explained by the fact that vegetarians tend to have better eating habits and be more concerned about health, leading to reduced consumption of processed foods such as soft drinks.

Regarding the average intake of natural and industrialized foods on the previous day, we observed that more than half (N = 6.98 of the 12 natural food groups listed were consumed with no significant variation among different types of vegetarians. On the other hand, from the 13 groups of industrialized foods listed, the average consumption was of 2.23 items (for all vegetarian categories), being lower in vegans than among the others (Table 4). The Dietary Guidelines for the Brazilian Population recommends that natural and fresh foods should be the basis of food intake, with a wide variety and most of the plant origin. On the other hand, the intake of some kinds of processed foods (such as white bread, and other refined or sugary-bakery products, cheeses, and canned foods) should be limited (or combined with healthy foods to compose a balanced diet), and highly processed food, including soft drinks, cookies, frozen meals, and chips, should be avoided, as they are nutritionally unbalanced [61].

The most consumed items from the list of natural foods were: cereals, legumes, and some of the vegetables (including green vegetables) and fruits items, being marked by over 80% of the individuals. On the other hand, meats, milk, and eggs were the less consumed items, which was expected, since milk and eggs are not suitable for vegans and meats (which includes fish) are consumed only by pesco- and semi-vegetarians. It is possible to suggest that the diet of most participants was based on cereals, legumes, fruits, and vegetables, which is in line with the main recommendations from the Dietary Guidelines for the Brazilian Population [61]. A recent systematic review published in 2019 evaluating the nutritional quality of vegetarian diets showed that vegetarians have a higher intake of fruits, whole grains, green vegetables, and legumes [62].

Regarding processed foods, the most consumed item included loaf bread, whole-grain loaf bread, hot dog bread, and burger bread. This category was related to bread types that are not homemade. However, despite being considered a processed food depending on how it is made, bread is traditionally part of the Brazilian diet. Moreover, depending on the diet context, bread can be inserted in a healthy diet, such as in the case of a Mediterranean diet, which is knowingly associated with improved cardiovascular and cognitive health, and prevention of diabetes, depression, obesity, some types of cancer, among other chronic diseases [63]. A review study on the Mediterranean diet constitution showed that the average bread consumption is of seven portions per day [64]. Moreover, a diet analysis of the EPIC-Oxford cohort participants showed that low-meat eaters (including all categories of vegetarians) consumed significantly less white bread and more whole-grain bread when compared to meat-eaters [65].

Since it is not possible to distinguish which type of bread was consumed by the participants of this study (white or whole-grain), more detailed analysis would be necessary to evaluate if this could be considered as a negative diet marker, or if it could be part of a balanced diet.

In the same category, the less consumed foods were processed meats. This result was also expected since these foods could be part of the semi-vegetarians diets only. Industrialized drinks (powder juice, boxed/canned juice, milk chocolate, soda, and flavored yogurt), margarine, and ready-to-eat meals were also among the less consumed foods. All these categories are considered highly processed foods by the Dietary Guidelines for the Brazilian Population and should be avoided because they are rich in fats and/or sugar, chemical additives, and sodium [61]. Results show that only a small percentage of the study participants consumed those items. These results are in line with EPIC-Oxford cohort data, which showed that, compared to meat-eaters, the low-meat eaters (including all categories of vegetarians) consumed lower amounts of refined grains, fried foods and sugar-sweetened beverages [65].

Comparison among data from the Brazilian general population and our study sample showed a small but significant difference regarding natural and processed foods intake. Vegetarians (all kinds) referred to an intake of a higher number of natural foods and a lower intake of processed foods. However, a greater difference in processed foods intake was observed mainly when comparing vegans (13.0% of items marked) with the Brazilian general population (17.2% of items marked). This result is in line with the one found by Clarys et al. [7], where vegans showed lower consumption of empty calories (considered as an unhealthy marker) and higher consumption of fruits and vegetables (considered as healthy markers) compared to omnivores. Ovo-lacto-vegetarians, pesco-vegetarians, and semi-vegetarians showed intermediate values.

Single item analysis showed that the Brazilian general population had a higher intake of soft drinks on the previous day when compared to vegetarians. This result is in line with the first part of the questionnaire, which showed that a lower proportion of vegetarians had a regular intake (five or more days per week) of soft drinks when compared to the Brazilian general population. Moreover, a broad view of the natural and processed foods intake analysis shows that vegetarians had a higher intake of most of the natural plant-based foods, such as some fruits, vegetables, whole grains, beans, and nuts. This result is also consistent with the highest proportion of vegetarians with a regular (five or more times per week) and adequate daily intake (five or more portions per day) of fruits and vegetables when compared to the Brazilian general population (Table 4).

When all variables are taken into account, it is possible to observe a trend of better results for vegans when compared to the other groups. Nonetheless, when all groups are analyzed together, results are still better than the ones observed in the general population in Brazil regarding the diet adequacy. A study conducted in Belgium to evaluate the nutritional quality of different types of vegetarians in comparison with omnivores showed similar results, with better diet quality indicators for vegans and worse results for omnivores, with intermediate results for ovo-lacto-vegetarians, pesco- and semi-vegetarians [7]. As shown by Parker & Vadiveloo (2019) on their systematic review, from the 12 studies included, results of nutritional quality were better in vegetarians than in nonvegetarians. Nutritional quality was measured with different diet quality indexes. Even though each index evaluates different items of the diet, the quality of the diet is always based on a combination of adequate components, such as fruits and vegetables, and moderation components, such as refined sugar [62].

Despite a general concern with nutritional deficiencies that might result from restrictive diets, vegetarian (including vegan) diets, if well planned, can be considered healthy and nutritionally adequate for all stages of life. Moreover, it can be adopted since early childhood and lead to healthy growth in comparison with an omnivore diet [66]. Vegetarianism is also associated with better health outcomes and the prevention of chronic diseases such as obesity, type 2 diabetes, hypertension, heart disease, and certain types of cancer [67].

The health benefits observed are associated with vegetarians’ higher intake of specific food groups, such as fruits, vegetables, and nuts, which are sources of phytochemicals, dietary fibers, and antioxidants and may, therefore, offer protection against chronic diseases [9]. Our study showed that vegetarian diets (including all categories) have better dietary markers when compared to the general Brazilian population. Diet quality was evaluated considering all markers used in *Vigitel*, which showed a higher percentage of individuals among vegetarians with regular weekly intake and adequate daily intake of fruits and vegetables, and a lower percentage of individuals with regular intake of soft drinks. Moreover, we observed an average intake of a wider variety of natural foods in comparison with industrialized foods, which is in line with the main recommendations of the Dietary Guidelines for the Brazilian Population [61].

Significant strengths of this study include the fact that we used a tool that is validated for the Brazilian population and has already been used in previous studies, allowing us to make comparisons among vegetarians, and between them and the general population in Brazil. Another strength is that we were able to recruit individuals from the entire country to participate, achieving a representative sample of the Brazilian vegetarian population, which enabled us to generate more consistent data in this area.

A limitation of this study might be the fact that our sample had a higher proportion of female respondents. Women had better results than men in some of the analyses. However, even the lowest results presented by males were higher than the ones from the Brazilian general population, in terms of regular and adequate intake of fruits and vegetables. Moreover, the same trend of better results for vegans compared to the other categories could be observed in both genders. Other studies with vegetarians also showed this trend, with over 70% of the sample being women [7,26]. According to Gossard & York [68], meat consumption is often associated with masculinity, and men tend to have more positive attitudes towards meat consumption when compared to women, who tend to consume less meat and appreciate plant-based foods because of its association to health. Moreover, women tend to be generally more concerned about the health and, therefore, participate more in health surveys than men [69,70], and this trend could explain the higher percentage of women found in our study.

Besides that, our sample was composed mostly of younger adults (below 40 years old), which could influence our results. However, is it possible that younger people are more prone to adopt a vegetarian diet, and that our results reflect, at least partially, the age distribution of vegetarians in Brazil. As observed in a cross-sectional analysis from the EPIC-Oxford cohort, with 65429 meat-eaters and non-meat-eaters, the median ages for men were 51, 49, 42, and 35 years, and for women 48, 39, 35 and 32 years for meat-eaters (omnivores), fish-eaters, vegetarians and vegans. In this study, participants had to be at least 20 years old [26]. It is possible to observe that, regarding animal product avoidance, the more restricted the diet, the lower was the median age, which could be a reality in Brazil as well.

It is important to address the fact that, due to different sampling methods, a comparison of our study sample with the Brasilian population was not adjusted for age, gender, or other variables. We understand that the different characteristics of both populations might partially explain the better results found among vegetarians. Nonetheless, comparisons made between vegetarians and the general Brazilian population were merely descriptive. Possible explanations of mechanisms and variables which could lead to these results can be explored in future studies.

Another potential limitation is that a convenience sample was used, which could result in the recruitment of more ‘health-conscious’ participants, which might limit the generalization of the results. On the other hand, a population-based study revealed that vegetarians appear to be more health-conscious when compared to non-vegetarians, showing that the positive results found in our study are not necessarily biased by the sampling method [71]. Moreover, random sampling would not allow us to attract enough participants, and therefore, it could make it more difficult for us to classify and compare different types of vegetarian diets. It is also important to point out that the questionnaire was not adapted to vegetarians on purpose, so it was possible to make comparisons between vegetarians and the general Brazilian population.

The method used to evaluate the dietary markers of the Brazilian vegetarian diet could also be considered a limitation of this study since we decided to use a tool adopted by the Brazilian government that only reflects some parameters of diet adequacy. Therefore, no nutrient intake was evaluated in this study, not allowing us to analyze specific nutrient deficiencies that could be associated with the vegetarian diet. On the other hand, due to the risk of bias associated with traditional diet report instruments, the use of brief tools might be a better approach to assess diet adequacy in broad population studies. Moreover, people do not eat single nutrients, but a combination of foods that interact and compose a dietary pattern, influencing health outcomes. Therefore, globally evaluating the diet quality, instead of using a single-nutrient approach, tend to be less biased when analyzing the correlation between diet and chronic diseases [72].

Conducting an online survey instead of face-to-face or phone interviews can also be a source of bias for the study since it limits the results to the individuals who have internet access. On the other hand, face-to-face interviews can also face difficulties in reaching a population parcel due to geographical limitations, which can be bypassed by using online surveys, which are also less invasive, costly, and time-consuming [73]. Moreover, data from the latest published research from the Brazilian Institute of Geography and Statistics (IBGE) showed that 3 out of 4 Brazilians have internet access, and the number of houses with landline dropped from 33,6% to 31,5%, while cellphone possession increased from 92.6% to 93.2% [74]. The cellphone was also the primary tool used to access the internet. Therefore, even though the online survey can be limited because it is not possible to reach a parcel of the population, it might still be a better strategy than phone interviews conducted through the landline. Our online survey could also be replied using a cellphone (not only computer), which helped to achieve more individuals.

Using self-reported intake can be a source of bias as well since it might lead to misreporting. However, there are no flawless methods for assessing diet intake, since other methods such as interviews and food records can result in the omission of food intake or behavioral change, which could also result in inaccurate intake estimates [75]. Therefore, we chose to use an online self-reported method because of its efficiency for data collection, positive impact on cost, and the possibility to reach a more significant number of participants.

Based on the results found in our study, it is possible that vegetarian diets could be seen as a potential strategy for public policies related to obesity control and improvement of population diet quality. Nonetheless, despite the positive results found in our study for vegetarians in comparison to the general Brazilian population, a large proportion of the participants does not achieve the fruits and vegetables daily intake, according to the World Health Organization recommendations. Therefore, public policies aimed at increasing fruit and vegetable intake in the population should also be targeted to vegetarians to improve diet quality in this group.

To our knowledge, this is the first study to assess the nationwide diet adequacy of vegetarian diets in Brazil. A systematic review published in 2019 about the nutritional quality of vegetarian diets showed that studies had been conducted in other countries, but none in Brazil or any Latin American country [62]. This study can open doors for more research to be done in the field of vegetarianism, to clarify the long-term effects of the vegetarian diet in health, as well as other factors that go beyond the diet quality, such as the potential impact on psychosocial aspects and quality of life.

## Conclusions

This is the first nationwide study to characterize the vegetarian population and its dietary markers. An overview of the diet adequacy showed that, based on the type of diet, a higher proportion of vegans showed positive results regarding diet quality, when compared to vegetarians, pesco-vegetarians, and semi-vegetarians. Diet quality markers included regular weekly intake and adequate daily intake of fruits and vegetables and low intake of soft drinks, as well as an analysis of specific food groups intake, divided into natural and processed foods. When analyzed together, vegetarians (all types) had better nutritional quality than the observed in Brazil for the general population. Data also showed that vegetarians seem to accomplish the main recommendations of the Dietary Guidelines for the Brazilian Population, with a higher intake of natural foods and a lower intake of processed foods, for all types of diets.

Nonetheless, despite these positive results found in our study, a large proportion of the participants does not achieve the fruits and vegetables daily intake, according to the World Health Organization recommendations. Therefore, public policies aimed at increasing fruit and vegetable intake in the population should also be targeted to vegetarians, to improve diet quality in this group.

This study can open doors for more research to be done in the field of vegetarianism, to clarify the long-term effects of the vegetarian diet in health, as well as other factors that go beyond the diet quality, such as the potential impact on psychosocial aspects and quality of life of Brazilian vegetarians.

## Supporting information

S1 Fig Graphic representation of the sample distribution according to Brazilian states and regions. Data from this study compared to data from *Mapaveg*.
(DOCX)

S2 Fig Comparison between results of diet adequacy and prevalence of overweight and obesity from this study with the Brazilian general population from *Vigitel* 2019 study (stratified by gender and age). (DOCX)

S1 Table VIGITEL 2018 food questionnaire in Portuguese. (DOCX)

S2 Table VIGITEL 2018 food questionnaire in English (free translation). (DOCX)

S3 Table Sample distribution according to Brazilian states and regions. Data from this study compared to data from *Mapaveg*.
(DOCX)

S4 Table Free translation of the natural and processed foods list, according to the *Vigitel* questions. (DOCX)
