# Correction: Brazilian vegetarians diet quality markers and comparison with the general population: A nationwide cross-sectional study

**DOI:** 10.1371/journal.pone.0235991

**Published:** 2020-07-06

**Authors:** Shila Minari Hargreaves, Wilma Maria Coelho Araújo, Eduardo Yoshio Nakano, Renata Puppin Zandonadi

There are a number of textual errors in the body of the article. The corrected sentences are provided below. The corrections do not change the overall conclusions. Please see the location of the error, the original text, and the author-corrected text here.

**Table:** 

**Location**	**Original text**	**Corrected text**
Materials and Methods, Procedures subsection, fourth paragraph, first sentence	“The diet quality results obtained in this study for vegetarians were compared with data from the last published version of the general Brazilian population study (Vigitel) [48].”	“The diet quality results obtained in this study for vegetarians were compared with data from the last published version of the general Brazilian population study (Vigitel) [50].”
Results, Population characteristics subsection, first sentence	“The majority (65.0%) were female, and 65.3% of individuals were below 40 years old.”	“The majority (65.0%) were female, and 65.7% of individuals were below 40 years old.”
Results, Population characteristics subsection, third sentence	“Pesco-vegetarian and semi-vegetarian diets had lower prevalence, accounting together for 13.4% of the sample.”	“Pesco-vegetarian and semi-vegetarian diets had lower prevalence, accounting together for 18.2% of the sample.”
Results, Diet quality regarding the adequacy to the Brazilian guideline subsection, fifth paragraph, fourth sentence	“The less consumed items were processed meats, artificial juice powder e ready-to-eat meals (instant noodles/soups, frozen lasagna, and other frozen ready-to-eat meals).”	“The less consumed items were processed meats, artificial juice powder, and ready-to-eat meals (instant noodles/soups, frozen lasagna, and other frozen ready-to-eat meals).”
Discussion, second paragraph, fourth sentence	“Different from our study, the authors directly weighted and heightened the participants; however, the sample was smaller than ours.”	“Different from our study, the authors directly weighted and measured the participants; however, the sample was smaller than ours.”
Discussion, seventh paragraph, third sentence	“A review study on vegetarian and low-meat diets showed that, indeed, vegetarian diets are characterized by the higher consumption of fruits, vegetables, and nuts, a factor that is associated with its protective effect against chronic diseases.”	“A review study on vegetarian and low-meat diets showed that, indeed, vegetarian diets are characterized by the higher consumption of fruits, vegetables, and nuts, a factor that is associated with their protective effect against chronic diseases.”
Discussion, ninth paragraph, third sentence	“The average percentage of individuals regularly consuming soft drinks was 3.9% in our study, a number significantly lower than the prevalence of 14.6% in the Brazilian population.”	“The average percentage of individuals regularly consuming soft drinks was 3.9% in our study, a number significantly lower than the prevalence of 14.4% in the Brazilian population.”
Discussion, ninth paragraph, sixth sentence	“By observing the trends from the last 11 years and the established projections for the general Brazilian population, it is possible to consider that the prevalence found in our study is very low among vegetarians, even when comparing the different categories separately, with the semi-vegetarians showing the highest prevalence among them (6.0%).”	“By observing the trends from the last 11 years and the established projections for the general Brazilian population, it is possible to consider that the prevalence found in our study is very low among vegetarians, even when comparing the different categories separately, with the ovo-lacto-vegetarians showing the highest prevalence among them (5.8%).”
Discussion, tenth paragraph, first sentence	“Regarding the average intake of natural and industrialized foods on the previous day, we observed that more than half (N = 6.98 of the 12 natural food groups listed were consumed with no significant variation among different types of vegetarians.”	“Regarding the average intake of natural and industrialized foods on the previous day, we observed that more than half (N = 6.98) of the 12 natural food groups listed were consumed with no significant variation among different types of vegetarians.”
Discussion, tenth paragraph, third sentence	“The Dietary Guidelines for the Brazilian Population recommends that natural and fresh foods should be the basis of food intake, with a wide variety and most of the plant origin.”	“The Dietary Guidelines for the Brazilian Population recommends that natural and fresh foods should be the basis of food intake, with a wide variety and most of plant origin.”
Discussion, eleventh paragraph, first sentence	“The most consumed items from the list of natural foods were: cereals, legumes, and some of the vegetables (including green vegetables) and fruits items, being marked by over 80% of the individuals.”	“The most consumed items from the list of natural foods were: cereals, legumes, and some of the vegetables (including green vegetables) and fruits items, being marked by over 75% of the individuals.”
Discussion, fifteenth paragraph, third sentence	“However, a greater difference in processed foods intake was observed mainly when comparing vegans (13.0% of items marked) with the Brazilian general population (17.2% of items marked).”	“However, a greater difference in processed foods intake was observed mainly when comparing vegans (13.0% of items marked) with the Brazilian general population (21.4% of items marked).”
Discussion, eighteenth paragraph, second sentence	“Moreover, it can be adopted since early childhood and lead to healthy growth in comparison with an omnivore diet [66].”	“Moreover, they can be adopted since early childhood and lead to healthy growth in comparison with an omnivore diet [66].”
Conclusions, first sentence	“This is the first nationwide study to characterize the vegetarian population and its dietary markers.”	“This is the first nationwide study to characterize the Brazilian vegetarian population and its dietary markers.”

There is an error in reference 50. The correct reference is: Ministério da Saúde. Vigitel Brasil 2018: Vigilância de fatores de risco e proteção para doenças crônicas por inquerito telefônico. Vigitel Bras 2018. Brasília; 2019. Available: http://portalarquivos2.saude.gov.br/images/pdf/2019/julho/25/vigitel-brasil-2018.pdf
