# Supplementary material for: Brazilian vegetarians diet quality markers and comparison with the general population: A nationwide cross-sectional study
Source: PLoS One. 2020 May 12;15(5):e0232954. doi: 10.1371/journal.pone.0232954 (PMC7217440; doi:10.1371/journal.pone.0232954)
Supplement: S1 Fig — Data from this study compared to data from Mapaveg. (DOCX) [file pone.0232954.s001.docx]

**S1 Fig:** **Graphic representation of the sample distribution according to Brazilian states and regions.** Data from this study compared to data from *Mapaveg*.
