# Supplementary material for: Brazilian vegetarians diet quality markers and comparison with the general population: A nationwide cross-sectional study
Source: PLoS One. 2020 May 12;15(5):e0232954. doi: 10.1371/journal.pone.0232954 (PMC7217440; doi:10.1371/journal.pone.0232954)

**S2 Fig:** Comparison between results of diet adequacy and prevalence of overweight and obesity from this study with the Brazilian general population from *Vigitel* 2019 study (Stratified by gender and age).


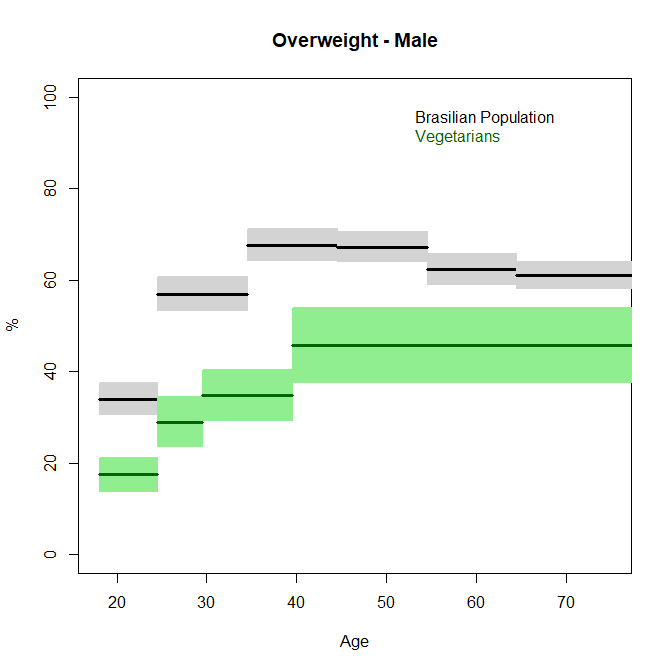

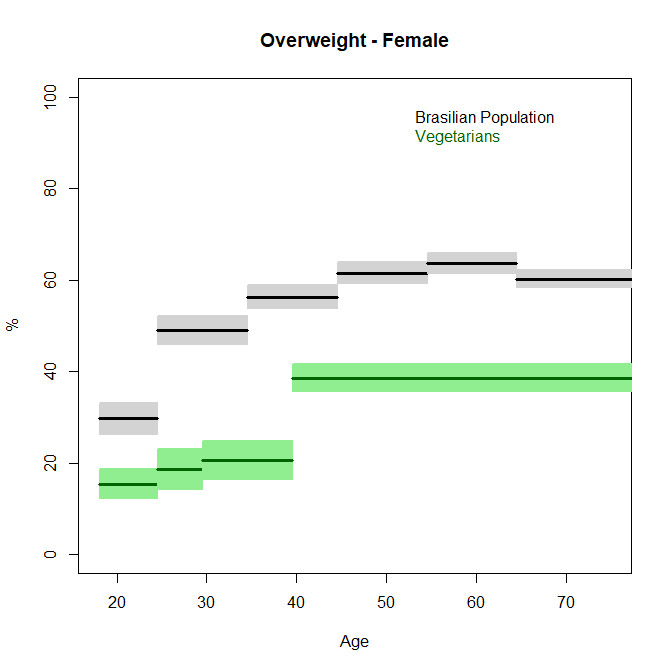


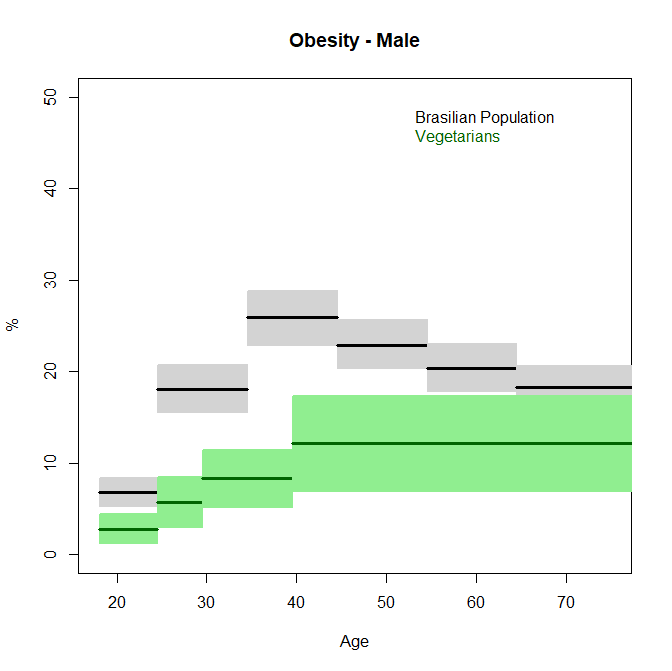

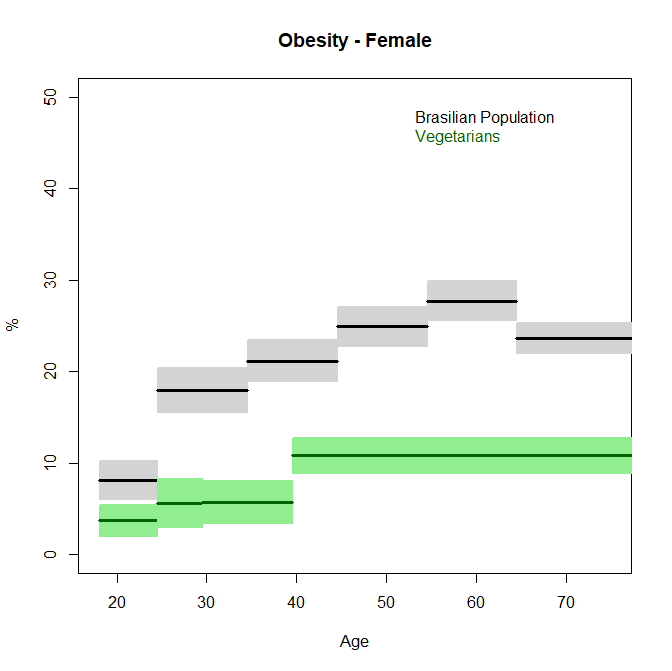


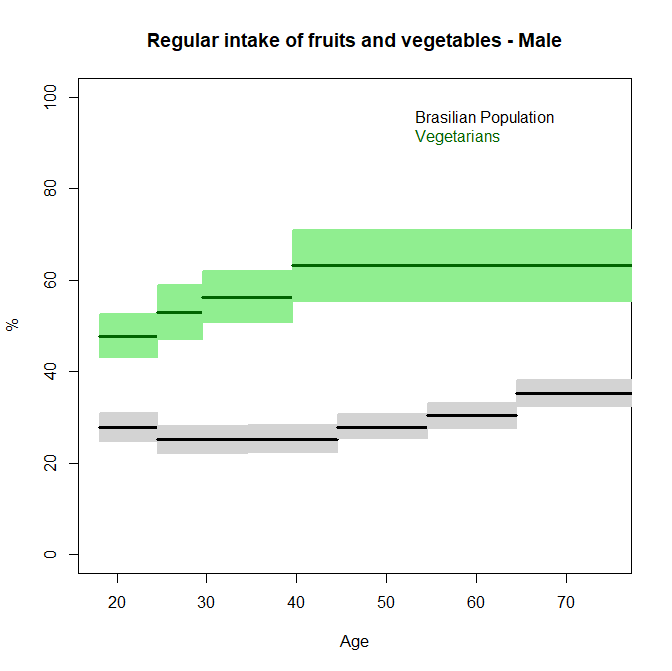

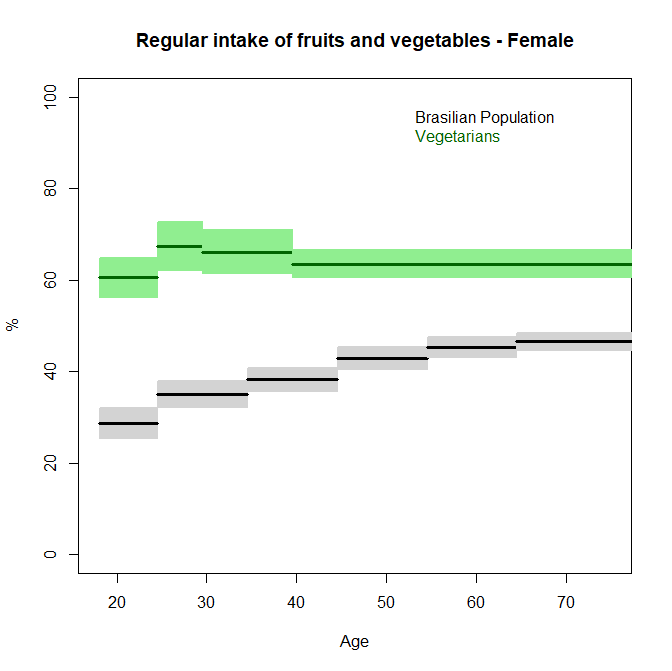


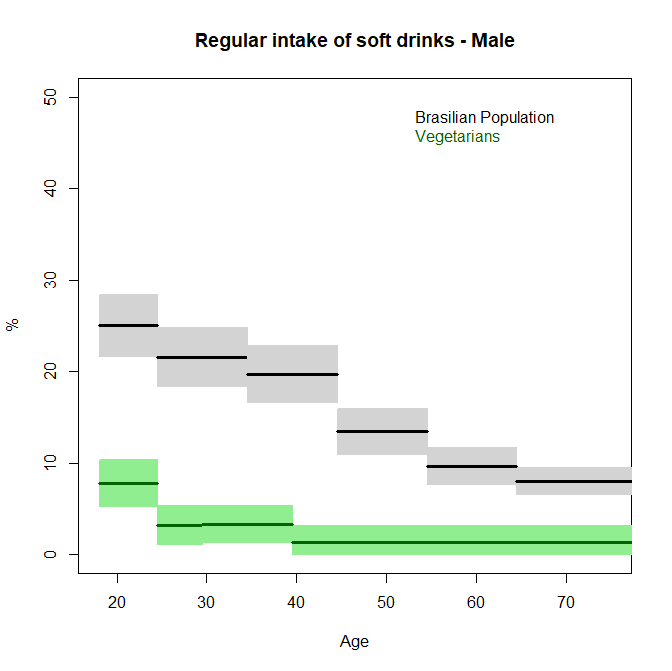

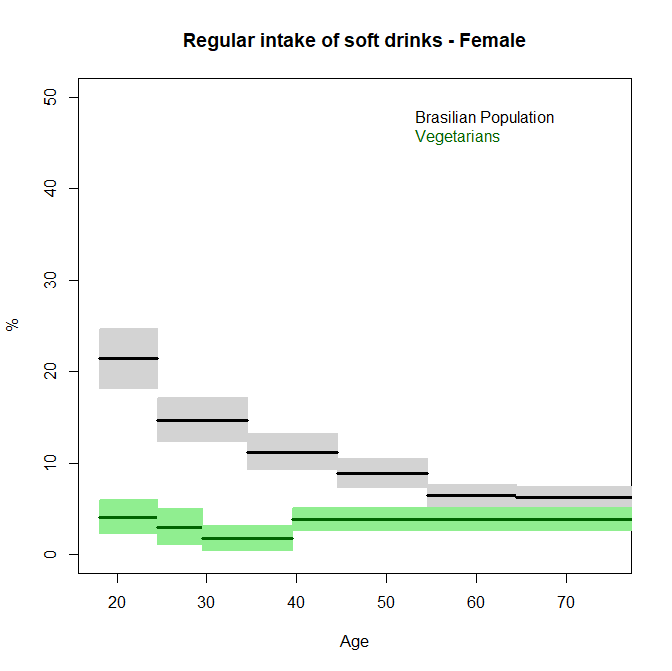


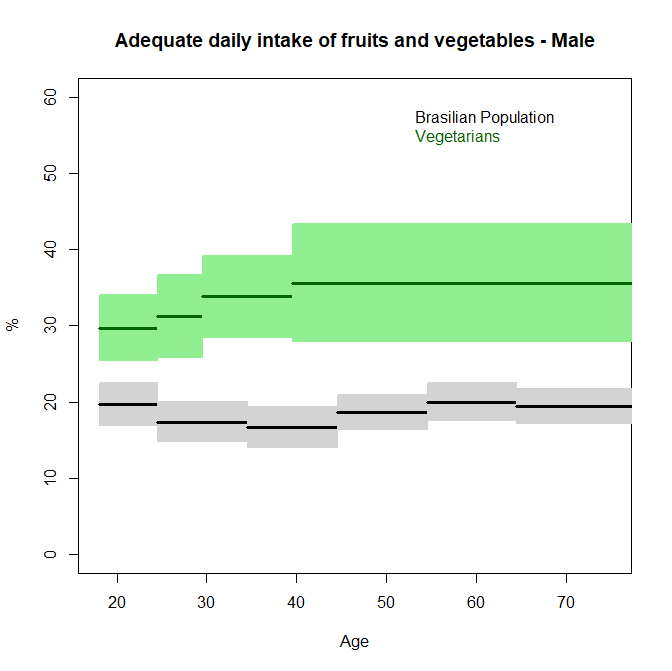

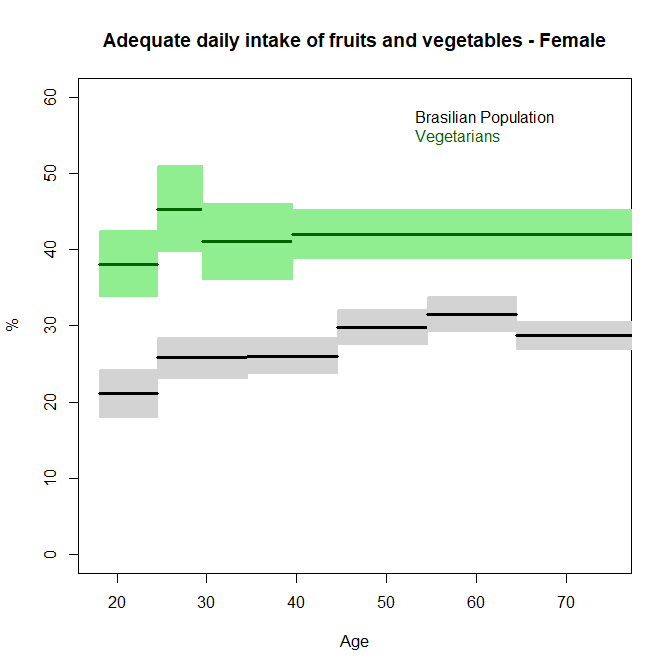

Supplement: S2 Fig — (DOCX) [file pone.0232954.s002.docx]
