# Supplementary material for: Brazilian vegetarians diet quality markers and comparison with the general population: A nationwide cross-sectional study
Source: PLoS One. 2020 May 12;15(5):e0232954. doi: 10.1371/journal.pone.0232954 (PMC7217440; doi:10.1371/journal.pone.0232954)
Supplement: S1 Table — (DOCX) [file pone.0232954.s003.docx]

S1 Table: VIGITEL 2018 food questionnaire in Brazilian-Portuguese.

| **Questionário do Vigitel 2018 para avaliar qualidade nutricional** | | | | | | |
| --- | --- | --- | --- | --- | --- | --- |
| **Q1. Em quantos dias da SEMANA você costuma comer pelo menos um tipo de verdura ou legume (ex: alface, tomate, couve, cenoura, chuchu, berinjela, abobrinha – não vale batata, mandioca ou inhame)?** | | | | | | |
| 1. 1 a 2 dias por semana  2. 3 a 4 dias por semana  3. 5 a 6 dias por semana  4. todos os dias (inclusive sábado e domingo)  5. quase nunca  6. nunca 🡪 **pule para Q6** | | | | | | |
| **Q2. Em quantos dias da SEMANA você costuma comer salada de alface e tomate ou salada de qualquer outra verdura ou legume CRU?** | | | | | | |
| 1. 1 a 2 dias por semana  2. 3 a 4 dias por semana  3. 5 a 6 dias por semana  4. todos os dias (inclusive sábado e domingo)  5. quase nunca 🡪 **pule para Q4**  6. nunca 🡪 **pule para Q4** | | | | | | |
| **Q3. Num dia comum, você come este tipo de salada:** | | | | | | |
| 1. no almoço (1 vez no dia) 2. no jantar ou 3. no almoço e no jantar (2 vezes no dia) | | | | | | |
| **Q4. Em quantos dias da SEMANA você costuma comer verdura ou legume COZIDO junto com a comida ou na sopa, como por exemplo, couve, cenoura, chuchu, berinjela, abobrinha (sem contar batata, mandioca ou inhame)?** | | | | | | |
| 1. 1 a 2 dias por semana  2. 3 a 4 dias por semana  3. 5 a 6 dias por semana  4. todos os dias (inclusive sábado e domingo)  5. quase nunca 🡪 **pule para Q6**  6. nunca 🡪 **pule para Q6** | | | | | | |
| **Q5. Num dia comum, você come verdura ou legume cozido:** | | | | | | |
| 1. no almoço (1 vez no dia) 2. no jantar ou 3. no almoço e no jantar (2 vezes no dia) | | | | | | |
| **Q6. Em quantos dias da SEMANA você costuma tomar suco de frutas natural (considere também polpa de fruta)?** | | | | | | |
| 1. 1 a 2 dias por semana  2. 3 a 4 dias por semana  3. 5 a 6 dias por semana  4. todos os dias (inclusive sábado e domingo)  5. quase nunca 🡪 **pule para Q8**  6. nunca 🡪 **pule para Q8** | | | | | | |
| **Q7. Num dia comum, quantos copos você toma de suco de frutas natural?** | | | | | | |
| 1. 1 2. 2 3. 3 ou mais | | | | | | |
| **Q8. Em quantos dias da SEMANA você costuma comer frutas?** | | | | | | |
| 1. 1 a 2 dias por semana  2. 3 a 4 dias por semana  3. 5 a 6 dias por semana  4. todos os dias (inclusive sábado e domingo)  5. quase nunca 🡪 **pule para Q10**  6. nunca 🡪 **pule para Q10** | | | | | | |
| **Q9. Num DIA comum, quantas vezes você come frutas?** | | | | | | |
| 1. 1 vez no dia 2. 2 vezes no dia 3. 3 ou mais vezes no dia | | | | | | |
| **Q10. Em quantos dias da SEMANA você costuma tomar refrigerante ou suco artificial?** | | | | | | |
| 1. 1 a 2 dias por semana  2. 3 a 4 dias por semana  3. 5 a 6 dias por semana  4. todos os dias (inclusive sábado e domingo)  5. quase nunca  6. nunca 🡪 **pule para Q13** | | | | | | |
| **Q11. Que tipo?** | | | | | | |
| 1. normal 2. diet/light/zero 3. ambos | | | | | | |
| **Q12. Quantos copos/latinhas costuma tomar por dia?** | | | | | | |
|  | 1. 1 2. 2 | 1. 3 2. 4 | 1. 5 2. 6 | 1. 7 ou mais 2. não sei | |  |
| ***Agora responda se você comeu algum destes alimentos ONTEM (desde quando acordou até quando foi dormir)*** | | | | | | |
|  | | | | | | |
| **Q13.** **Alimentos naturais** ou **básicos** | | | | | SIM | NÃO |
| **a.** Alface, couve, brócolis, agrião ou espinafre  **b.** Abóbora, cenoura, batata-doce ou quiabo/caruru  **c.** Mamão, manga, melão amarelo ou pequi  **d.** Tomate, pepino, abobrinha, berinjela, chuchu ou beterraba  **e.** Laranja, banana, maçã ou abacaxi  **f.** Arroz (considere também arroz integral), macarrão, polenta ou angu, cuscuz ou milho verde  **g.** Feijão, ervilha, lentilha ou grão de bico  **h.** Batata comum, mandioca (considere farinha de mandioca ou tapioca), cará ou inhame  **i.** Carne de boi, porco, frango ou peixe (considere vísceras; não considere embutidos, hambúrguer, *nuggets,* salsicha e produtos similares).  **j.** Ovo frito, cozido ou mexido (considere omelete; não considere ovos em preparações e massas)  **k.** Leite (não considere leites vegetais)  **l.** Amendoim, castanha de caju ou castanha do Brasil/Pará | | | | |  |  |
| **Q14. Agora vou relacionar alimentos ou produtos industrializados.** | | | | | | |
| **a.** Refrigerante  **b.** Suco de fruta em caixa, caixinha ou lata  **c.** Refresco em pó  **d.** Bebida achocolatada  **e.** Iogurte com sabor  **f.** Salgadinho de pacote (ou chips) ou biscoito/bolacha salgado (considerar também os integrais)  **g.** Biscoito/bolacha doce, biscoito recheado ou bolinho de pacote  **h.** Chocolate, sorvete, gelatina, flan ou outra sobremesa industrializada (não considerar balas, pirulitos e chicletes)  **i.** Salsicha, linguiça, mortadela ou presunto  **j.** Pão de forma (mesmo o integral), de cachorro-quente ou de hambúrguer  **k.** Maionese, ketchup ou mostarda  **l.** Margarina  **m.** Macarrão instantâneo, sopa de pacote, lasanha congelada ou outro prato pronto comprado congelado | | | | |  |  |
