# Supplementary material for: Brazilian vegetarians diet quality markers and comparison with the general population: A nationwide cross-sectional study
Source: PLoS One. 2020 May 12;15(5):e0232954. doi: 10.1371/journal.pone.0232954 (PMC7217440; doi:10.1371/journal.pone.0232954)
Supplement: S2 Table — (DOCX) [file pone.0232954.s004.docx]

S2 Table: VIGITEL 2018 food questionnaire in English (free translation).

| **Vigitel 2018 questionnaire to evaluate the nutritional quality** | | | | | |  |
| --- | --- | --- | --- | --- | --- | --- |
| **Q1. In how many days of the WEEK you usually eat at least one type of vegetable (ex: lettuce, tomato, kale, carrot, chayote, eggplant, zucchini – potato, manioc or yam does not count)?** | | | | | |  |
| 1. once to twice per week  2. tree to four times per week  3. five to six times per week  4. every day (including Saturday and Sunday)  5. almost never  6. never 🡪 **skip to Q6** | | | | | |  |
| **Q2. In how many days of the WEEK do you usually eat a salad with lettuce and tomatoes or any other type of RAW vegetable?** | | | | | |  |
| 1. once to twice per week  2. tree to four times per week  3. five to six times per week  4. every day (including Saturday and Sunday)  5. almost never 🡪 **skip to Q4**  6. never 🡪 **skip to Q4** | | | | | |  |
| **Q3. In a typical day, do you eat this type of salad:** | | | | | |  |
| 1. at lunch (once per day) 2. at dinner 3. at lunch and dinner (twice per day) | | | | | |  |
| **Q4. In how many days of the WEEK do you usually eat a COOKED vegetable with your meal or in the soup, such as kale, carrot, chayote, eggplant, zucchini (potato, manioc or yam does not count)?** | | | | | |  |
| 1. once to twice per week  2. tree to four times per week  3. five to six times per week  4. every day (including Saturday and Sunday)  5. almost never 🡪 **skip to Q6**  6. never 🡪 **skip to Q6** | | | | | |  |
| **Q5. In a common day, do you eat cooked vegetables:** | | | | | |  |
| 1. at lunch (once per day) 2. at dinner 3. at lunch and dinner (twice per day) | | | | | |  |
| **Q6. In how many days of the WEEK do you usually drink natural fruit juice (consider also pulp fruit juice)?** | | | | | |  |
| 1. once to twice per week  2. tree to four times per week  3. five to six times per week  4. every day (including Saturday and Sunday)  5. almost never 🡪 **skip to Q8**  6. never 🡪 **skip to Q8** | | | | | |  |
| **Q7. In a common day, how many glasses of natural fruit juice do you drink?** | | | | | |  |
| 1. 1 2. 2 3. 3 or more | | | | | |  |
| **Q8. In how many days of the WEEK do you usually eat fruits?** | | | | | |  |
| 1. once to twice per week  2. tree to four times per week  3. five to six times per week  4. every day (including Saturday and Sunday)  5. almost never 🡪 **skip to Q10**  6. never 🡪 **skip to Q10** | | | | | |  |
| **Q9. In a common DAY, how many times do you eat fruits?** | | | | | |  |
| 1. Once per day 2. Twice per day 3. Three or more times per day | | | | | |  |
| **Q10. In how many days of the WEEK do you usually dring soda or artificial juice?** | | | | | |  |
| 1. once to twice per week  2. tree to four times per week  3. five to six times per week  4. every day (including Saturday and Sunday)  5. almost never  6. never 🡪 **skip to Q13** | | | | | |  |
| **Q11. Of which kind?** | | | | | |  |
| 1. regular 2. diet/light/zero 3. both | | | | | |  |
| **Q12. How many cups/cans do you usually drink per day?** | | | | | |  |
|  | 1. 1 2. 2 | 1. 3 2. 4 | 1. 5 2. 6 | 1. 7 or more 2. I don’t know | |  |
| ***Now answer if you ate any of these foods YESTERDAY (from the time you woke up until you went to bed)*** | | | | | |  |
|  | | | | | |  |
| **Q13.** **Natural** **foods** | | | | | SIM | NÃO |
| **a.** Lettuce, kale, broccoli, cress, or spinach  **b.** Pumpkin, carrot, sweet potato, or okra  **c.** Papaya, mango, melon, or pequi  **d.** Tomato, cucumber, zucchini, eggplant, chayote or beetroot  **e.** Orange, banana, apple or pineapple  **f.** Rice (consider also brown rice), pasta, cooked cornmeal, couscous or corn  **g.** Beans, peas, lentils, or chickpeas  **h.** Potato, manioc (consider manioc flour or tapioca flour) or yams  **i.** Beef, pork, chicken, or fish (consider viscera; do not consider processed meats, burgers, nuggets*,* sausages, and similar products).  **j.** Egg – fried, boiled or scrambled (consider omelet; do not consider eggs used as ingredients in dishes and doughs)  **k.** Milk (do not consider plant-based milk)  **l.** Peanut, cashew nut or Brazil nut | | | | |  |  |
| **Q14. Processed foods** | | | | | |  |
| **a.** Soda  **b.** Boxed or canned juice  **c.** Artificial juice powder  **d.** Chocolate milk  **e.** Flavored yogurt  **f.** Chips or crackers (consider also whole-grain crackers)  **g.** Sweet biscuits/cookies, sandwich cookies or industrialized cakes  **h.** Chocolate, ice cream, gelatin, flan or other industrialized desserts (do not consider candy, lollipop and chewing gum)  **i.** Sausage, mortadella, or ham  **j.** Loaf bread (even whole grain), hot dog bread, or burger bread  **k.** Mayonnaise, ketchup, or mustard  **l.** Margarine  **m.** Instant noodles/soup, frozen lasagna or other frozen instant meals | | | | |  |  |
