# Supplementary material for: Brazilian vegetarians diet quality markers and comparison with the general population: A nationwide cross-sectional study
Source: PLoS One. 2020 May 12;15(5):e0232954. doi: 10.1371/journal.pone.0232954 (PMC7217440; doi:10.1371/journal.pone.0232954)
Supplement: S3 Table — Data from this study compared to data from Mapaveg. (DOCX) [file pone.0232954.s005.docx]

**S3 Table: Sample distribution according to Brazilian states and regions.** Data from this study compared to data from *Mapaveg*.

|  | **MapaVeg** | **Study** |  |  | **MapaVeg** | **Study** |
| --- | --- | --- | --- | --- | --- | --- |
| **State** | **freq (%)** | **freq (%)** |  | **Region*** | **freq (%)** | **freq (%)** |
| Distrito Federal | 1470 (5.02%) | 394 (11.87%) |  | MW | 2512 (8.58%) | 508 (15.31%) |
| Goiás | 523 (1.79%) | 58 (1.75%) |  |  |  |  |
| Mato Grosso | 176 (0.6%) | 22 (0.66%) |  |  |  |  |
| Mato Grosso do Sul | 291 (0.99%) | 30 (0.90%) |  |  |  |  |
| Tocantins | 52 (0.18%) | 4 (0.12%) |  |  |  |  |
| Acre | 24 (0.08%) | 1 (0.03%) |  | N | 443 (1.51%) | 43 (1.30%) |
| Amapá | 20 (0.07%) | 2 (0.06%) |  |  |  |  |
| Amazonas | 125 (0.43%) | 17 (0.51%) |  |  |  |  |
| Pará | 205 (0.7%) | 17 (0.51%) |  |  |  |  |
| Rondônia | 68 (0.23%) | 3 (0.09%) |  |  |  |  |
| Roraima | 1 (0%) | 3 (0.09%) |  |  |  |  |
| Alagoas | 136 (0.46%) | 22 (0.66%) |  | NE | 2904 (9.92%) | 354 (10.67%) |
| Bahia | 731 (2.5%) | 93 (2.8%) |  |  |  |  |
| Ceará | 504 (1.72%) | 64 (1.93%) |  |  |  |  |
| Maranhão | 92 (0.31%) | 10 (0.30%) |  |  |  |  |
| Paraíba | 291 (0.99%) | 28 (0.84%) |  |  |  |  |
| Pernambuco | 620 (2.12%) | 76 (2.29%) |  |  |  |  |
| Piauí | 104 (0.36%) | 10 (0.30%) |  |  |  |  |
| Rio Grande do Norte | 294 (1%) | 30 (0.90%) |  |  |  |  |
| Sergipe | 132 (0.45%) | 21 (0.63%) |  |  |  |  |
| Paraná | 2267 (7.74%) | 190 (5.72%) |  | S | 7222 (24.66%) | 640 (19.28%) |
| Rio Grande do Sul | 2946 (10.06%) | 250 (7.53%) |  |  |  |  |
| Santa Catarina | 2009 (6.86%) | 200 (6.03%) |  |  |  |  |
| Espírito Santo | 364 (1.24%) | 49 (1.48%) |  | SE | 16201 (55.33%) | 1774 (53.45%) |
| Minas Gerais | 2504 (8.55%) | 310 (9.34%) |  |  |  |  |
| Rio de Janeiro | 2968 (10.14%) | 310 (9.34%) |  |  |  |  |
| São Paulo | 10365 (35.4%) | 1105 (33.29%) |  |  |  |  |
| **TOTAL** | **29282** | **3319** |  |  |  |  |

*MD: Midwest; N: North; NE: Northeast; S: South; SE: Southeast.

Source: Mapaveg ^46^.
