# Supplementary material for: Brazilian vegetarians diet quality markers and comparison with the general population: A nationwide cross-sectional study
Source: PLoS One. 2020 May 12;15(5):e0232954. doi: 10.1371/journal.pone.0232954 (PMC7217440; doi:10.1371/journal.pone.0232954)
Supplement: S4 Table — (DOCX) [file pone.0232954.s006.docx]

**S4 Table: Free translation of the natural and processed foods list, according to the *Vigitel* questions.**

| **Natural foods** |
| --- |
| **a.** Lettuce, kale, broccoli, cress, or spinach  **b.** Pumpkin, carrot, sweet potato, or okra  **c.** Papaya, mango, melon, or pequi  **d.** Tomato, cucumber, zucchini, eggplant, chayote or beetroot  **e.** Orange, banana, apple or pineapple  **f.** Rice (consider also brown rice), pasta, cooked cornmeal, couscous or corn  **g.** Beans, peas, lentils, or chickpeas  **h.** Potato, manioc (consider manioc flour or tapioca flour) or yams  **i.** Beef, pork, chicken, or fish (consider viscera; do not consider processed meats, burgers, nuggets*,* sausages, and similar products).  **j.** Egg – fried, boiled or scrambled (consider omelet; do not consider eggs used as ingredients in dishes and doughs)  **k.** Milk (do not consider plant-based milk)  **l.** Peanut, cashew nut or Brazil nut |
| **Processed foods** |
| **a.** Soda  **b.** Boxed or canned juice  **c.** Artificial juice powder  **d.** Chocolate milk  **e.** Flavored yogurt  **f.** Chips or crackers (consider also whole-grain crackers)  **g.** Sweet biscuits/cookies, sandwich cookies, or industrialized cakes  **h.** Chocolate, ice cream, gelatin, flan or other industrialized desserts (do not consider candy, lollipop and chewing gum)  **i.** Sausage, mortadella, or ham  **j.** Loaf bread (even whole grain), hot dog bread, or burger bread  **k.** Mayonnaise, ketchup, or mustard  **l.** Margarine  **m.** Instant noodles/soup, frozen lasagna or other frozen instant meals |
